# Reconstruction of knee anatomy from single-plane fluoroscopic x-ray based on a nonlinear statistical shape model

**DOI:** 10.1117/1.JMI.8.1.016001

**Published:** 2021-01-11

**Authors:** Jing Wu, Mohamed R. Mahfouz

**Affiliations:** University of Tennessee, Department of Mechanical, Aerospace, and Biomedical Engineering, Knoxville, Tennessee, United States

**Keywords:** 2D–3D non-rigid registration, 3D shape reconstruction, kernel principal component analysis, fluoroscopic x-ray, statistical shape model, total joint replacement

## Abstract

**Purpose:** Reconstruction of patient anatomy is critical to patient-specific instrument (PSI) design in total joint replacement (TJR). Conventionally, computed tomography (CT) and magnetic resonance imaging (MRI) are used to obtain the patient anatomy as they are accurate imaging modalities. However, computing anatomical landmarks from the patient anatomy for PSIs requires either high-resolution CT, increasing time of scan and radiation exposure to the patient, or longer and more expensive MRI scans. As an alternative, reconstruction from single-plane fluoroscopic x-ray provides a cost-efficient tool to obtain patient anatomical structures while allowing capture of the patient’s joint dynamics, important clinical information for TJR.

**Approach:** We present a three-dimensional (3D) reconstruction scheme that automatically and accurately reconstructs the 3D knee anatomy from single-plane fluoroscopic x-ray based on a nonlinear statistical shape model called kernel principal component analysis. To increase robustness, we designed a hybrid energy function that integrated feature and intensity information as a similarity measure for the 3D reconstruction.

**Results:** We evaluated the proposed method on five subjects during deep knee bending: the root-mean-square accuracy is 1.19±0.36  mm for reconstructed femur and 1.15±0.17  mm for reconstructed tibia.

**Conclusions:** The proposed method demonstrates reliable 3D bone model reconstruction accuracy with successful elimination of prior 3D imaging and reduction of manual labor and radiation dose on patient as well as characterizing joints in motion. This method is promising for applications in medical interventions such as patient-specific arthroplasty design, surgical planning, surgical navigation, and understanding anatomical and dynamic characteristics of joints.

## 1 Introduction

Reconstruction of patient anatomy from medical images is important for designing patient-specific instruments (PSIs) for total joint replacement (TJR).^1^^–^^4^ Current 3D reconstruction methods include reconstruction from radiological images using computed tomography (CT), magnetic resonance imaging (MRI),^5^^,^^6^ x-ray,^7^^–^^9^ and fluoroscopic x-ray sequences.^10^^,^^11^ Three-dimensional reconstruction from high-resolution CT is expensive and requires prior 3D imaging, exposing patients to large amounts of radiation (0.01 rem for lower extremities) by revolving the x-ray source around the patient with a fine “fan” of x-ray beams through the body from all angles into their associated detectors.^12^ Three-dimensional reconstruction from MRI requires expensive and time-consuming 3D imaging, adding discomfort to the patient in the form of noise and constraint to the patient’s motion. In addition, reconstruction from CT or MRI requires significant manual intervention for 3D segmentation. Although reconstruction from x-ray images is more cost-efficient than CT or MRI, the x-ray image is static, and therefore, gives limited dynamic information of the joint for clinical assessment.

In contrast, three-dimensional reconstruction from fluoroscopic x-ray sequences provides a clear advantage in lower cost and neglectable radiation (0.003 rem for ∼33  s) and involves less manual intervention compared with reconstruction from CT and MRI.^12^ In addition, fluoroscopic x-ray images are sequences of x-ray images that can be captured during dynamic, weight-bearing activities, whereas x-ray imaging is static. Single-plane fluoroscopic x-ray is a useful tool for analyzing joint anatomy and joint kinematics *in vivo*,^13^^,^^14^ because it allows sufficiently unconstrained motion of the patients, such as deep knee bending (DKB), which can reveal soft tissue information of the joint in multiple positions. Biplanar x-ray using two orthogonal units, although generally more accurate, often unacceptably constrains the motion of the patient.^15^ In order to capture kinematic information, we reconstruct the 3D anatomy of the knee from single-plane fluoroscopic x-ray to allow patients to move without impairment.

Three-dimensional knee reconstruction from kinematic sequences was first proposed by Baker et al.^10^ However, this requires volumetric image reconstruction by scanning 360 deg around the patient. Baka et al.^7^ then reconstructed a distal femur from stereo x-ray images. The method was not optimized to sequence reconstruction as bone shape is expected to stay constant throughout the sequence. An improvement was made by the same author^11^ to estimate the shape based on all frames of the fluoroscopic x-ray sequence and estimate pose per frame using biplane fluoroscopic x-ray. We propose to reconstruct patient knee anatomy during kinematic motion from single-plane fluoroscopic x-ray sequences employing a two-stage optimization scheme. In the first stage, the pose is estimated with the mean model; in the second stage, optimization alternates between shape and pose. This two-stage optimization is challenging due to the limited information provided from single-plane images and large motion between frames. To address this, we employ a statistical shape model (SSM) to have good shape representation ability and a robust hybrid similarity measure.

Methods for bone shape reconstruction have been proposed in the literature with statistical shape modeling. The SSM was first introduced by Cootes et al. in 1995 who used “shape priors” to restrict the bone reconstruction to plausible shapes.^16^ The linear SSM is “learned” by a principal component analysis (PCA) of the training shapes of Gaussian-distributed shapes. Although this model has been successfully applied to the 3-D reconstruction of various anatomical structures in medical imaging,^7^^,^^11^^,^^17^ there are cases when the set of training shapes exhibits highly nonlinear shape deformations, such as large shape variations between bones. More recently, other shape reconstruction methods include Gaussian process morphable models (GPMMs)^18^ and an articulated SSM.^19^ Lüthi et al.^18^ developed GPMMs to represent the shape variations with a Gaussian process using the leading components of its Karhunen–Loeve expansion. The method reaches its limitations when very fine deformations need to be modeled over a large domain, as is sometimes required in image registration. Balestra et al.^19^ reconstructed 3-D patient-specific hip joints from x-ray images. In this work, an articulated statistical shape model was utilized to address the problems associated with hip joint narrowing. An average reconstruction error of 1.9 mm was achieved for the hip and 1.1 mm was achieved for the proximal femur.

In this paper, we build on our previously developed method for creation of statistical atlases and establishing correspondence between anatomical models.^20^^–^^23^ In order to accommodate for the nonlinear variations, we employ kernel PCA (KPCA)^24^^,^^25^ to map the original data from the input space to a high-dimensional feature space via a nonlinear map and then apply a linear PCA in the feature space. (The justification analysis of the need for a nonlinear SSM is detailed in the Appendix.)

The main contributions of this paper are threefold. (1) We propose a 3-D reconstruction scheme from single-plane fluoroscopic x-ray sequences during kinematic activity. The 3-D reconstruction is based on kernel PCA as a nonlinear SSM to represent the bone shape variation. (2) We propose a hybrid energy function as the similarity measure to integrate feature and intensity information in the fluoroscopic x-ray images. (3) We perform a multi-body reconstruction of knee bones (e.g., distal femur and proximal tibia) from single-plane fluoroscopic x-ray during kinematic activity.

From a clinical point of view, the proposed method offers valuable information needed for accurate assessment of the knee joint in preoperative planning. First, the reconstruction of the patient anatomy under load-bearing conditions provides more accurate representation of the joint alignment compared to the nonload-bearing supine position in CT and MRI. Second, collecting patient kinematic information under load-bearing conditions offers critical information about behavior of soft tissue during unconstrained patient motion. This overcomes the current limitation of kinematic data collection in knee replacement candidates, which is currently obtained using surgical navigation systems intraoperatively and using passive motion of the patient joint under anesthesia. In summary, the reconstructed models, joint alignment, and kinematic information that are obtained can lead to more accurate preoperative planning and execution using either patient-specific instrumentation or computer/robotic guided surgery.

## 2 Approach

**Fig. 1:**
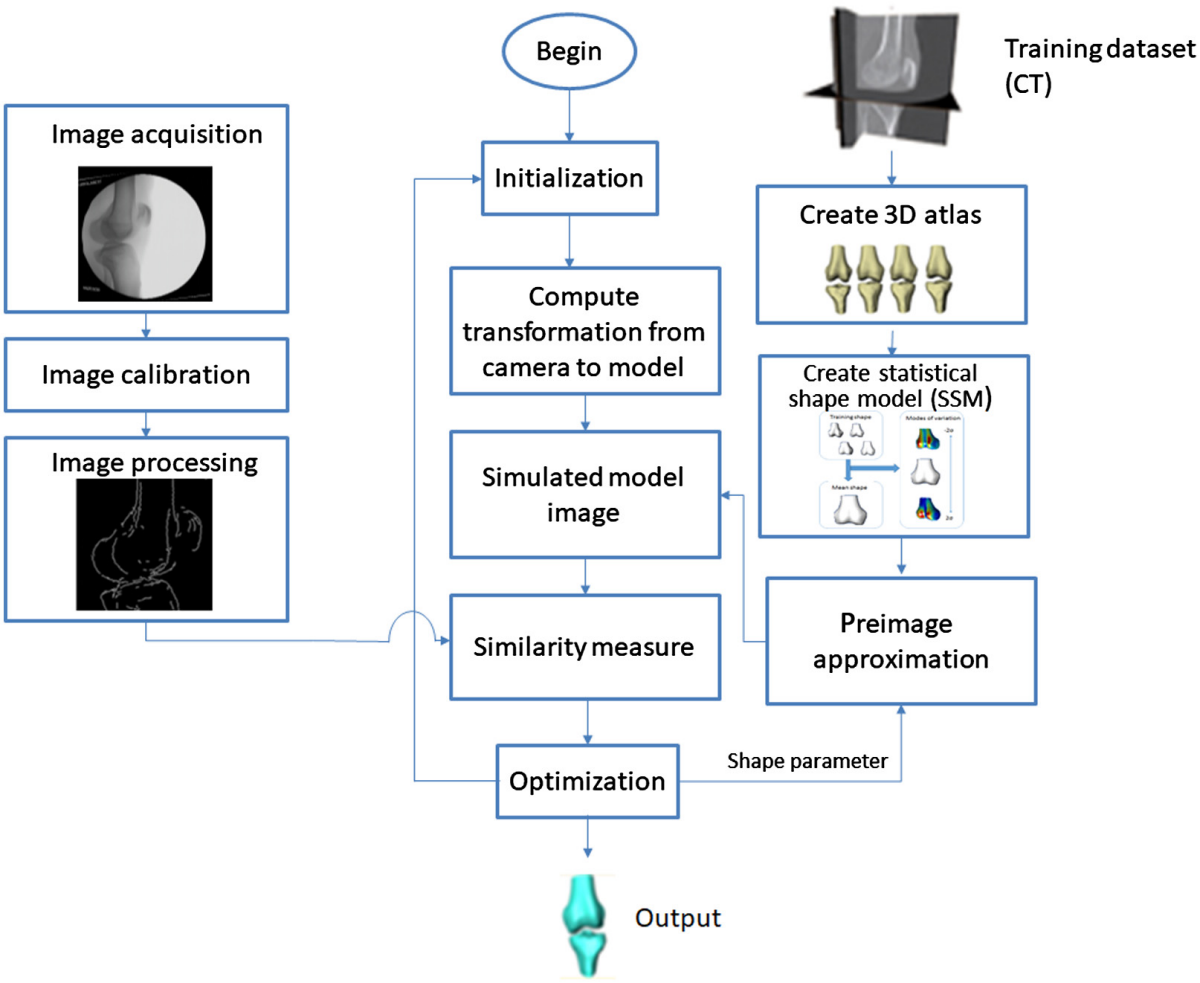
Framework of the proposed method.

The goal of this paper is to reconstruct the 3-D knee anatomy from a single-plane fluoroscopic x-ray sequence. The outline of the general framework is shown in Fig. 1. First, digital video fluoroscopic x-ray [two-dimensional (2-D)] images are acquired, allowing capture of dynamic data during kinematic activities. Each frame is calibrated using a known rectangular grid of metal beads.^13^ The pose of the 3-D model is initialized with a template matching method^26^ and the shape is initialized as a mean model. The 3-D model is reconstructed from a nonlinear SSM using KPCA. This requires a training process and preimage approximation as described in Sec. 2.1. The reconstruction employs a two-stage optimization scheme for the pose and shape of the -3-D model as discussed in Sec. 2.2. A hybrid energy function as discussed in Sec. 2.3 is used as a similarity measure. The output is the patient-specific knee 3-D model with poses corresponding to each frame in the fluoroscopic x-ray sequence.

### 2.1 Nonlinear Statistical Shape Model

The creation of statistical atlases and establishing correspondence between anatomical models previously developed by our group is a verified method utilized in multiple medical imaging applications.^20^^–^^23^ The addition described in this paper is the use of kernel PCA to capture nonlinear variations in anatomical structures (Sec. 2.1.1) and then performing a preimage approximation (Sec. 2.1.2) to get the reconstructed 3-D model from shape parameters. A set of 300 mixed gender knee bones of Caucasian and black American individuals were used to build this SSM.

#### 2.1.1 SSM training by kernel principal component analysis

The SSM represents the dominant shape variation of the training set. The training data $X\in R^{3}$ are 3-D surface mesh models defined as $X=\{x_{i}|i\in \{1,\ldots ,N\}\}$, where N is the number of training data. Each training shape $x_{i}\subset X$ has a set of vertices, where $x_{i}=\{x_{i}^{k}|k\in \{1,\ldots ,M\}\}$, M denotes the number of vertices in the 3-D surface mesh model, and $x_{i}^{k}=(x,y,z)$ denotes the coordinates of the k’th vertex of $x_{i}$. Corresponding points are found between each training shape before building the SSM with a statistical shape atlas.^20^^–^^23^

The input data are mapped onto a high-dimensional feature space H via the nonlinear map $\Phi :R^{3}\to H$. This map does not need to be explicitly known. Alternatively, one can introduce the kernel matrix K, which is defined to be a function as follows:

$$K(x_{i},x_{j})=\langle \Phi (x_{i}),\Phi (x_{j})\rangle ,$$ (1)

such that for all data points $x_{i}$

$$K=(\begin{array}{ccc} k(x_{1},x_{1}) & \cdots & k(x_{1},x_{N})\\ \vdots & \ddots & \vdots \\ k(x_{N},x_{1}) & \ldots & k(x_{N},x_{N}) \end{array})$$

is symmetric and positive semidefinite. For a Gaussian kernel

$$K(x_{i},x_{j})=e^{-\frac{{\left\|x_{i}-x_{j}\right\|}^{2}}{2\sigma^{2}}}.$$

Center $\Phi (x_{i})$ by $\Phi (x_{i})≔\Phi (x_{i})-\frac{1}{N}\overset{N}{\underset{i=1}{\sum }}\Phi (x_{i})$. The corresponding kernel matrix is modified as follows:

$${\overset{¯}{K}}_{ij}=K-\frac{1}{N}\underset{j}{\sum }K_{ij}-\frac{1}{N}\underset{i}{\sum }K_{ij}+\frac{1}{N^{2}}\underset{i}{\sum }\underset{j}{\sum }K_{ij}.$$ (2)

Conduct PCA by solving the eigenvalue problem

CV=λV, (3)

where $C=\frac{1}{N}\overset{N}{\underset{i=1}{\sum }}\Phi (x_{i})\Phi {\left(x_{i}\right)}^{T}$, V are the eigenvectors, and λ are the eigenvalues. Since the eigenvectors V lie in the span of $\Phi (x_{i}),\ldots ,\Phi (x_{N})$, Eq. (3) is equivalent to

$$\Phi (x_{i})\cdot \overset{¯}{C}V=\lambda [\Phi (x_{i})\cdot V]\;\text{for all}\;i=1,\ldots ,N.$$ (4)

Since $K(x_{i},x_{j})=\langle \Phi (x_{i}),\Phi (x_{j})\rangle$ is symmetric and it has a set of eigenvectors that spans the whole space, this is equivalent to solving the dual eigenvalue problem

Kα=Nλα, (5)

where α are the eigenvectors and λ is the eigenvalue for both problems. Then standard PCA is conducted to get the first m eigenvectors to constitute the eigenmatrix A.

Normalize the eigenvectors $\alpha^{1},\ldots ,\alpha^{N}$ by requiring that the corresponding vectors in H be normalized, i.e., $\langle V^{k},V^{k}\rangle =1$ for all k=1,…,N. This is equivalent to

$$1=\overset{N}{\underset{i,j=1}{\sum }}\alpha_{i}^{k}\alpha_{j}^{k}\Phi (x_{i})\cdot \Phi (x_{j})=\lambda_{k}(\alpha^{k}\cdot \alpha^{k}).$$ (6)

Project the input data $X\in R^{3}$ onto the feature space spanned by the first m eigenvectors of C. The projected input data are given by

$$P^{m}\Phi (X)=\langle V^{T},\Phi (X)\rangle =\overset{m}{\underset{i=1}{\sum }}\alpha_{i}\Phi (X_{i})\Phi (X)=\overset{m}{\underset{i=1}{\sum }}\alpha_{i}K(X_{i},X).$$ (7)

#### 2.1.2 Preimage approximation

We need to reconstruct the preimages X^ from the shape parameter $\theta =\{\theta_{1},\ldots ,\theta_{m}\}$ in this section. In standard PCA, the preimage X^ can simply be approximated by the linear relationship between the shape parameters and the preimage.^16^ However, this simple linear relationship does not exist for KPCA because the nonlinear map Φ is not necessarily known.^27^ For kernel PCA, we reconstruct the preimage X^ of the corresponding test point $X\in R^{3}$ based on the relationship between input-space and feature-space distances.^25^

For any two points $x_{i}$ and $x_{j}$ in the input space, the Euclidean distance is given by $d(x_{i},x_{j})$. Accordingly, their feature-space distance $\tilde{d}[\Phi (x_{i}),\Phi (x_{j})]$ is obtained using the mapped images. The relationship between $d(x_{i},x_{j})$ and $\overset{˜}{d}[\Phi (x_{i}),\Phi (x_{j})]$ can be derived as follows:

$${\overset{˜}{d}}^{2}[\Phi (x_{i}),\Phi (x_{j})]=1-\alpha_{1}K\alpha_{1}-2K\alpha_{1},$$ (8)

where $\alpha_{1}=\alpha (\theta -b)$. For the Gaussian kernel of the form $k(\Phi_{i},\Phi_{j})=e^{-\frac{{\left\|x_{i}-x_{j}\right\|}^{2}}{2\sigma^{2}}}$, which is invertible, the relationship between $d(x_{i},x_{j})$ and $\tilde{d}[\Phi (x_{i}),\Phi (x_{j})]$ can be described by

$$d_{ij}^{2}=-2\sigma^{2}\;\log[\frac{1}{2}(K_{ii}+K_{jj}-{\overset{˜}{d}}_{ij}^{2})].$$ (9)

The distance to neighboring points has an exponential impact on the estimation of the current point according to the iterative scheme defined by Schölkopf et al.^28^ The contribution of neighboring points drops rapidly with increasing distance from the preimage, and only n neighbors $\{x_{1},\ldots ,x_{n}\}\in R^{M}$ are obtained, where n≤N.

An M×n matrix is constructed, $X=[x_{1},\ldots ,x_{n}]$. Assuming that the training patterns span an m-dimensional space, we can obtain the singular value decomposition of the centered M×n matrix XH as

$$XH=U\Lambda V^{\prime }=UZ,$$ (10)

where the left singular vector $U=[e_{1},\ldots e_{m}]$ is an M×m matrix with orthonormal columns $e_{i}$ and Λ is the right singular vector, Λ are singular values, and Z is an m×n matrix with columns $z_{i}$ containing the projection of $x_{i}$ onto $e_{j}^{\prime }\;s$. The squared distance of $x_{i}$ to the origin is equal to ${\left\|z_{i}\right\|}^{2}$ such that $d_{0}^{2}={\left[{\left\|z_{1}\right\|}^{2},\ldots ,{\left\|z_{n}\right\|}^{2}\right]}^{\prime }$.

According to the assumption that the preimage x^ is in the span of n neighbors, its location can be estimated by its n nearest neighbors. Hence the least squares solution z^ is yielded by

z^=−12Λ−1V′(d2−d02). (11)

The least squares solution z^ is expressed by the basis $e_{j}$ that defined a different coordinate system from the input space. Transforming it back to the original coordinate system x^

x^=Uz^+x¯. (12)

**Fig. 2:**
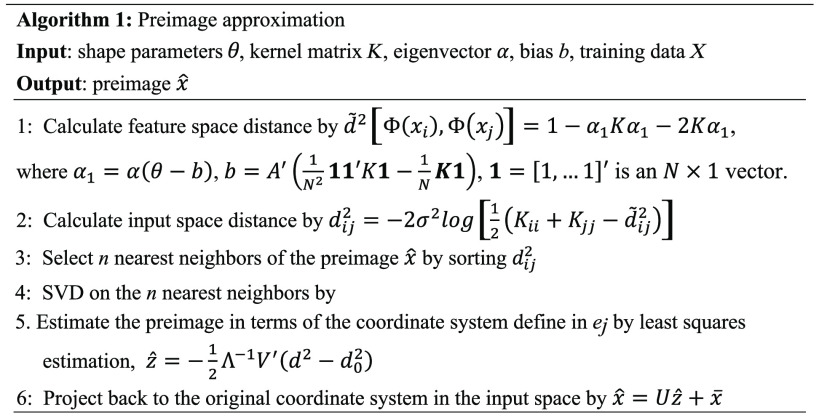
Preimage approximation algorithm.

This preimage approximation algorithm is summarized in Fig. 2.

### 2.2 Optimization

**Fig. 3:**
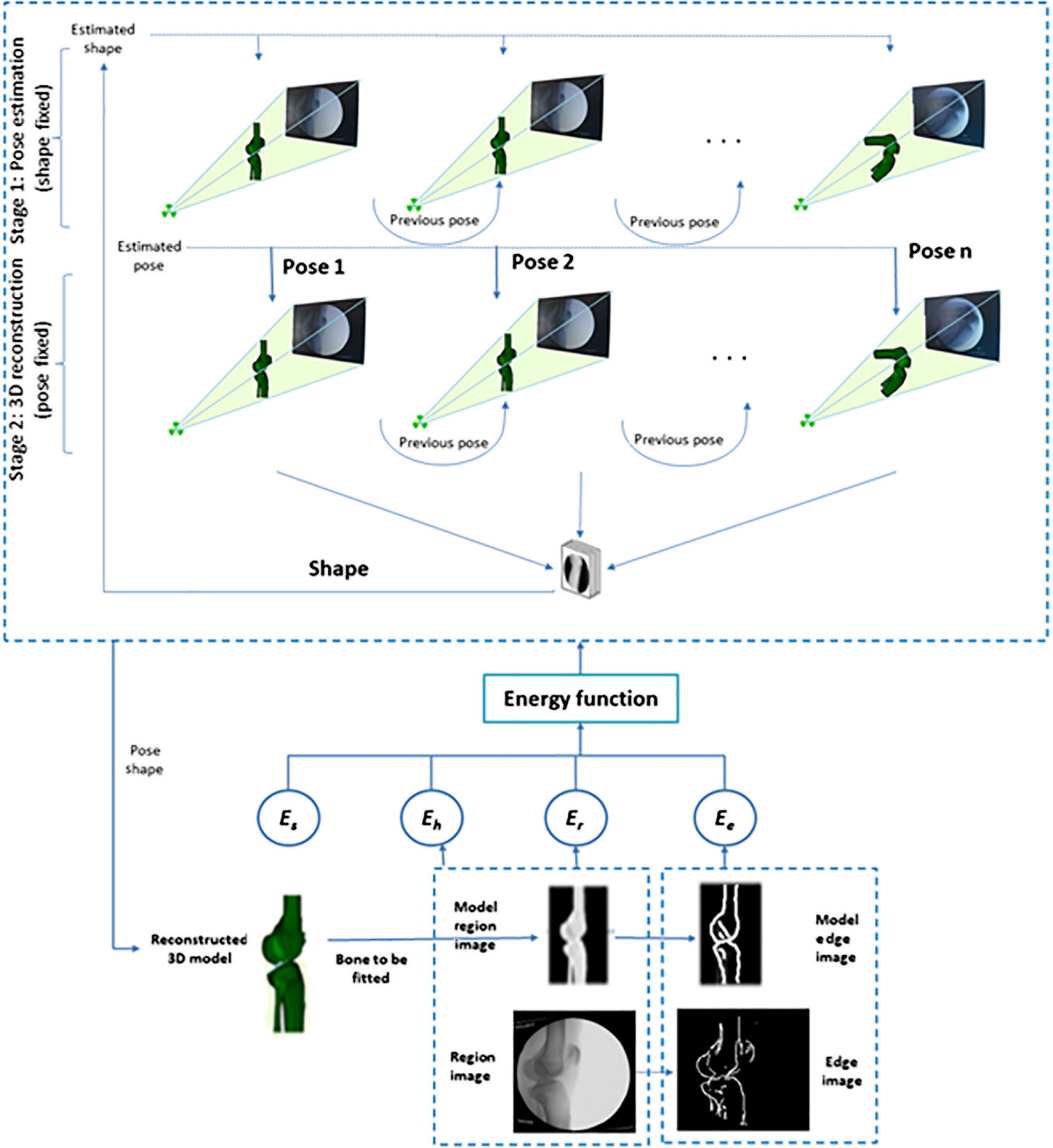
Two-stage optimization. Top: stage 1: the estimated shape is rigidly aligned to each frame of fluoroscopic x-ray image. Mean shape is used as initial shape parameter. Center: stage 2: shape is optimized with the fixed pose from stage 1. Stage 1 (pose) and stage 2 (shape) alternate optimization until convergence occurs. Bottom: The hybrid energy function is minimized to get the final estimation of the 3D model’s shape and pose.

To cope with the fitting of multiple parameters, we divided the optimization into two stages. The first stage provides an initialization for the reconstruction by fixing the shape parameters and optimizing pose parameters only. The initial fixed shape is the mean shape, which is rigidly registered to the 2-D image to yield a close-to-optimal pose. The second stage is shape estimation with the estimated pose from the first stage. These two stages run in turn, resulting in the optimal shape and pose estimation. The optimization scheme is shown in Fig. 3.

**Fig. 4:**
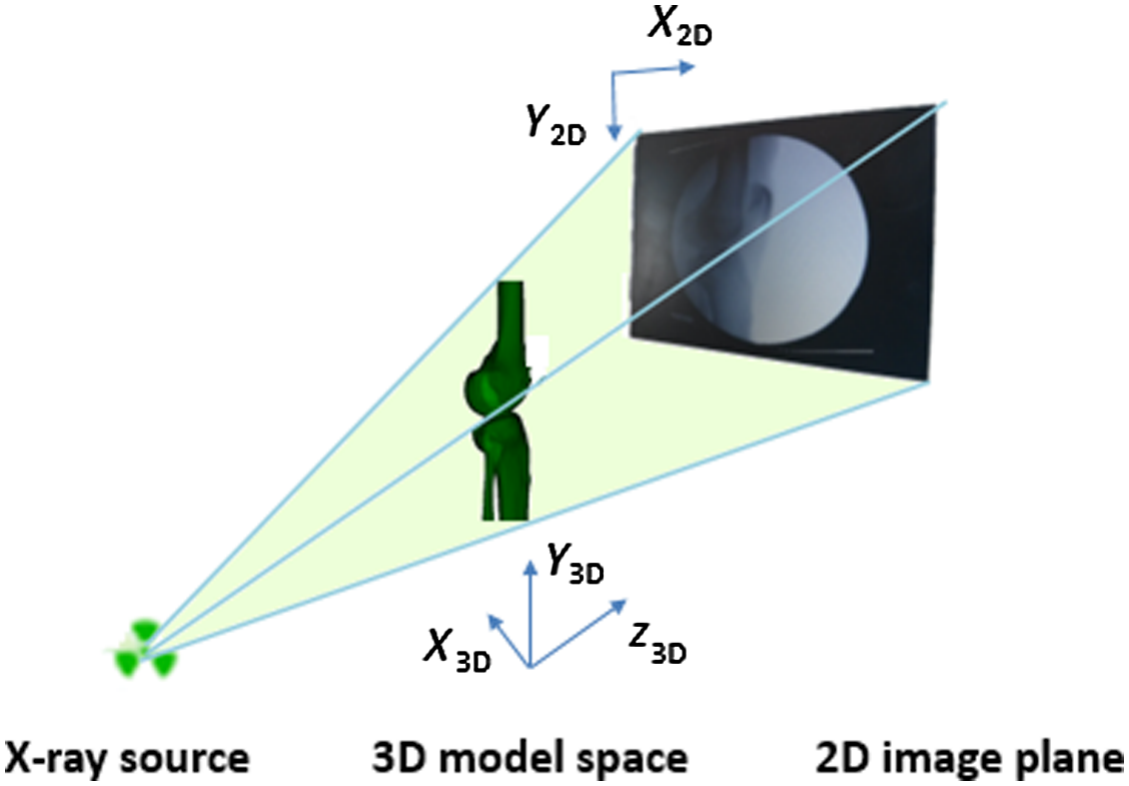
Camera coordinate system definition. In the sagittal view, x axis is around the AP axis, y axis is around the around the proximal-distal axis, and z axis is around the medial-lateral axis.

The shape and pose of the 3-D model are optimized in each frame $I_{i}$ of the fluoroscopic x-ray sequence. Let $S\in R^{3}$ be the smooth surface of the object of interest and denote X=[x,y,z], X∈S to be the spatial coordinates that are measured with respect to the referential frame of the imaging camera as shown in Fig. 4. Let $X_{0}\in R^{3}$ and $S_{0}\in R^{3}$ be the initial coordinates and surface in the camera reference frame, respectively. We assume a camera realization $\pi :R^{3}\to \Omega$ such that $X^{\prime }=[\frac{x}{z},\frac{y}{z}]$ and $\Omega \in R^{2}$ denotes the domain of the image $I_{i}$. Similarly, we can form the edge image of the object of interest in the image $I_{i}$ as $C_{i}=\partial I_{i}$, where $C_{i}\in R^{2}$.

We can locate the surface of the object of interest S in the camera reference frame via a transformation T such that $S=T(S_{0})$ and the corresponding pointwise expression is

$$X=T(X_{0})=RX_{0}+t,$$ (13)

where R is a rotation matrix. $R=R_{\gamma }R_{\beta }R_{\alpha }$ where

$$R_{\gamma }=[\begin{array}{ccc} \cos\;\gamma & -\sin\;\gamma & 0\\ \sin\;\gamma & \cos\;\gamma & 0\\ 0 & 0 & 1 \end{array}],\;R_{\beta }=[\begin{array}{ccc} \cos\;\beta & 0 & \sin\;\beta \\ 0 & 1 & 0\\ -\sin\;\beta & 0 & \cos\;\beta \end{array}],\;\;R_{\alpha }=[\begin{array}{ccc} 1 & 0 & 0\\ 0 & \cos\;\alpha & -\sin\;\alpha \\ 0 & \sin\;\alpha & \cos\;\alpha \end{array}]$$

and t is a translation vector, $t=[\begin{array}{c} t_{x}\\ t_{y}\\ t_{z} \end{array}]$.

X0=x^ is reconstructed from the shape parameters $\theta =\{\theta_{1},\ldots ,\theta_{m}\}$ via $X_{0}=f(\theta )$ using kernel PCA, which is described in Sec. 2.1.2.

Two sets of parameters are determined to align the 3-D model to the 2-D x-ray image: one is the shape parameter set $\theta =\{\theta_{1},\ldots ,\theta_{m}\}$; the other set contains the pose parameters, $p=\{\alpha ,\beta ,\gamma ,t_{x},t_{y},t_{z}\}$. The optimal shape and pose parameters are determined by minimizing the hybrid energy function defined in Sec. 2.3 with a global optimization algorithm called pattern search (PS). PS attempts to minimize an objective function by comparing its value in a finite set of trial points at each iteration. As a direct search method, PS can be applied to functions that are not continuous or differentiable. Convergence for stationary points can be proved from arbitrary starting points.^29^

### 2.3 Hybrid Energy Function

An energy function E, as a similarity measure, quantifies how closely the 3-D model with the current pose and shape fits the corresponding object in the fluoroscopic x-ray image. A hybrid energy function that integrates both feature and intensity information is proposed, which is less time consuming than the intensity-based method and less prone to segmentation error than the feature-based method. A collision detection score is used to avoid a collision between neighboring bones for multi-body registration. Specifically, the hybrid energy function is defined as a linear combination of an edge score $E_{e}$, a region score $E_{r}$, a homogeneity score $E_{h}$, and a collision detection score $E_{c}$

$$E=-[c_{1}u(x)E_{e}+c_{2}u(x)E_{r}-c_{3}E_{h}-c_{4}E_{c}],$$ (14)

where $c_{i}$ are the weighting parameters that set the contribution of each term to the overall energy function and u(x) is a mask. The value of u(x)=0 for pixels whose projection falls in the area of neighboring bones; u(x)=1 otherwise. The weighting scores are selected with the edge matching value weighted more heavily than the intensity matching values. By weighting the edge score more heavily than the intensity value, the edge value dominates when the 3-D models are close to the true contours. The weight of the homogeneity score is a penalty that matches the value of intensity and edge score, whereas the collision detection weight is a large constant to avoid such behavior.

Two input data are involved in the hybrid energy function: one is a 2-D x-ray image defined as $I_{1}:\Omega_{1}\in R^{2}$; the other is the projected 3-D model image defined as I2=π(x^), $I_{2}:\Omega_{2}\in R^{2}$. The generation of edge images is through a bilateral filter followed by a Canny edge detector. The corresponding edge images can be defined by $C_{1}=\partial I_{1}$ and $C_{2}=\partial I_{2}$, where $C_{1}$ is the edge image of the 2-D fluoroscopic x-ray image, $C_{2}$ is the projected 3-D model edge image, and $C_{1}$, $C_{2}\in R^{2}$.

The intensity information is integrated into the hybrid energy function by matching the x-ray image against the projected 3-D model image. Unlike intensity-based methods, which require a time-consuming digitally reconstructed radiograph (DRR) as the simulated x-ray projection image, the projected model image is generated by rendering the 3-D surface model on the 2-D image plane by software developed by the authors using a 3-D graphics library.^13^

The region score $E_{r}$ is thus defined as a modified local cross correlation^30^ as follows:

$$E_{r}(I_{1},I_{2})=\frac{1}{N}\underset{j\in \Omega_{1}}{\sum }\frac{\underset{i\in n(j)}{\sum }G_{p}(j){\left[I_{1}(i)-{\overset{¯}{I}}_{1}(j)\right]}^{2}{\left[I_{2}(i)-{\overset{¯}{I}}_{2}(j)\right]}^{2}}{\underset{i\in n(j)}{\sum }{\left[I_{1}(i)-{\overset{¯}{I}}_{1}(j)\right]}^{2}\underset{i\in n(j)}{\sum }{\left[I_{2}(i)-{\overset{¯}{I}}_{2}(j)\right]}^{2}},$$ (15)

where $I_{1}$ is the 2-D fluoroscopic x-ray image, $I_{2}$ is the projected 3-D model image, ${\overset{¯}{I}}_{1}$ and ${\overset{¯}{I}}_{2}$ are the corresponding mean values, i represents a pixel in the neighborhood n(j) around pixel j in $I_{1}$, and $G_{p}$ is a Gaussian window function centered around each pixel in the region image.

The feature information is integrated into the hybrid energy function by a direct image-to-image similarity measure in our previous work on rigid 2D–3D registration of knee implants^13^ as the edge score $E_{e}$ defined as follows:

$$E_{e}(C_{1},C_{2})=\frac{\sum_{i\in \Omega_{1},j\in \Omega_{2}}C_{1}(i)C_{2}(j)}{\sum_{j\in \Omega_{2}}C_{2}(j)}.$$ (16)

The edge score is maximized when the projected 3-D model edge image coincides with the corresponding object edges in the 2-D x-ray image.

The homogeneity score $E_{h}$ is defined as

$$E_{h}(I_{1},I_{2})=\underset{i\in C_{\text{in}}}{\sum }\frac{\underset{j\in n(i)}{\sum }|I_{1}(j)-{\overset{¯}{I}}_{\text{in}}(i)|}{n_{\text{in}}}+\underset{i\in C_{\text{out}}}{\sum }\frac{\underset{j\in n(i)}{\sum }|I_{1}(j)-{\overset{¯}{I}}_{\text{out}}(i)|}{n_{\text{out}}},$$ (17)

where the first term designates the variance of the set of gray levels located on the internal of the projected 3-D model image, and the second term designates the variance located on the external of the projected 3-D model. The parameter j represents a pixel in the neighborhood n(i) around pixel i in the 2-D x-ray image, ${\overset{¯}{I}}_{\text{in}}(i)$ is the mean intensity in the neighborhood of n(i) inside the 3-D model, $n_{\mathrm{in}}$ is the number of pixels inside the 3-D model, ${\overset{¯}{I}}_{\text{out}}(i)$ is the mean intensity in the neighborhood of n(i) outside the 3-D model, and $n_{\text{out}}$ is the number of pixels outside the 3-D model. $E_{h}$ is minimal when the external contour of the projected 3-D model delineates two homogeneous regions separated by an edge.

Neighboring bones may overlap each other during registration. Therefore, we include a collision detection score $E_{c}$ as a penalty to prevent collision of neighboring bones in the same frame

$$E_{c}(s_{1},s_{2})=CH_{1}(s_{1},s_{2}),$$ (18)

where C is a large constant, $s_{1}$ and $s_{2}$ are the surface mesh of the two involved bones, and $H_{1}$ is the Heaviside function used to penalize any overlapping between two surface models

$$H_{1}(s_{1},s_{2})=\{\begin{array}{ll} 1, & \text{if collision occurs}\\ 0, & \text{else} \end{array}.$$

Thus, if no collision is present, $H_{1}(s_{1},s_{2})$ will be equal to zero.

**Fig. 5:**
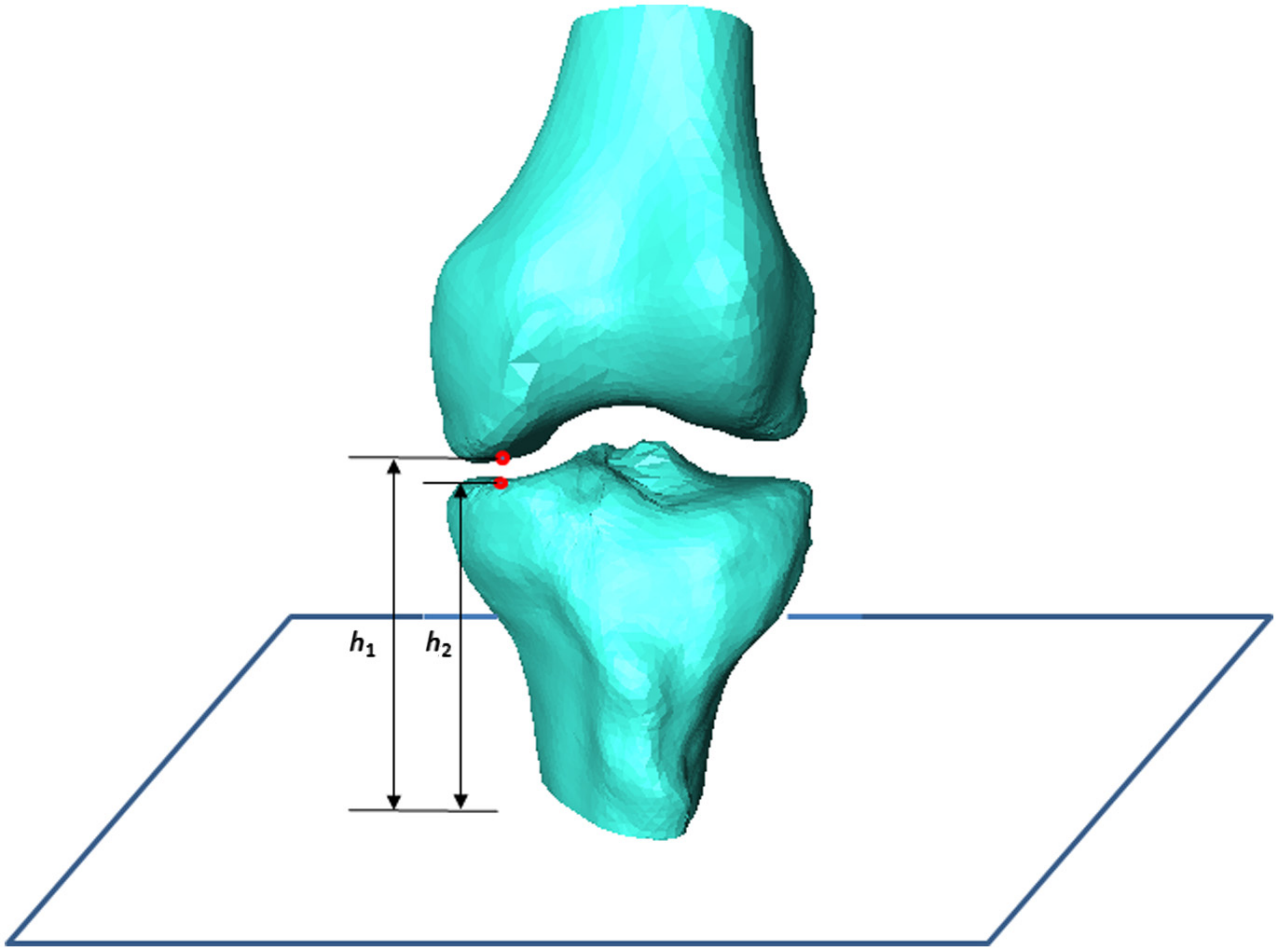
Collision detection by comparing the distances to tibia bottom plane.

**Fig. 6:**
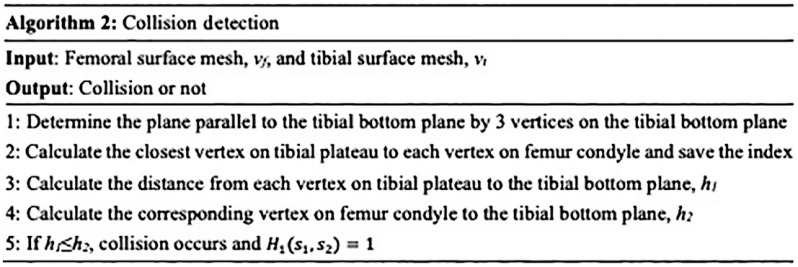
Collision detection algorithm.

**Fig. 7:**
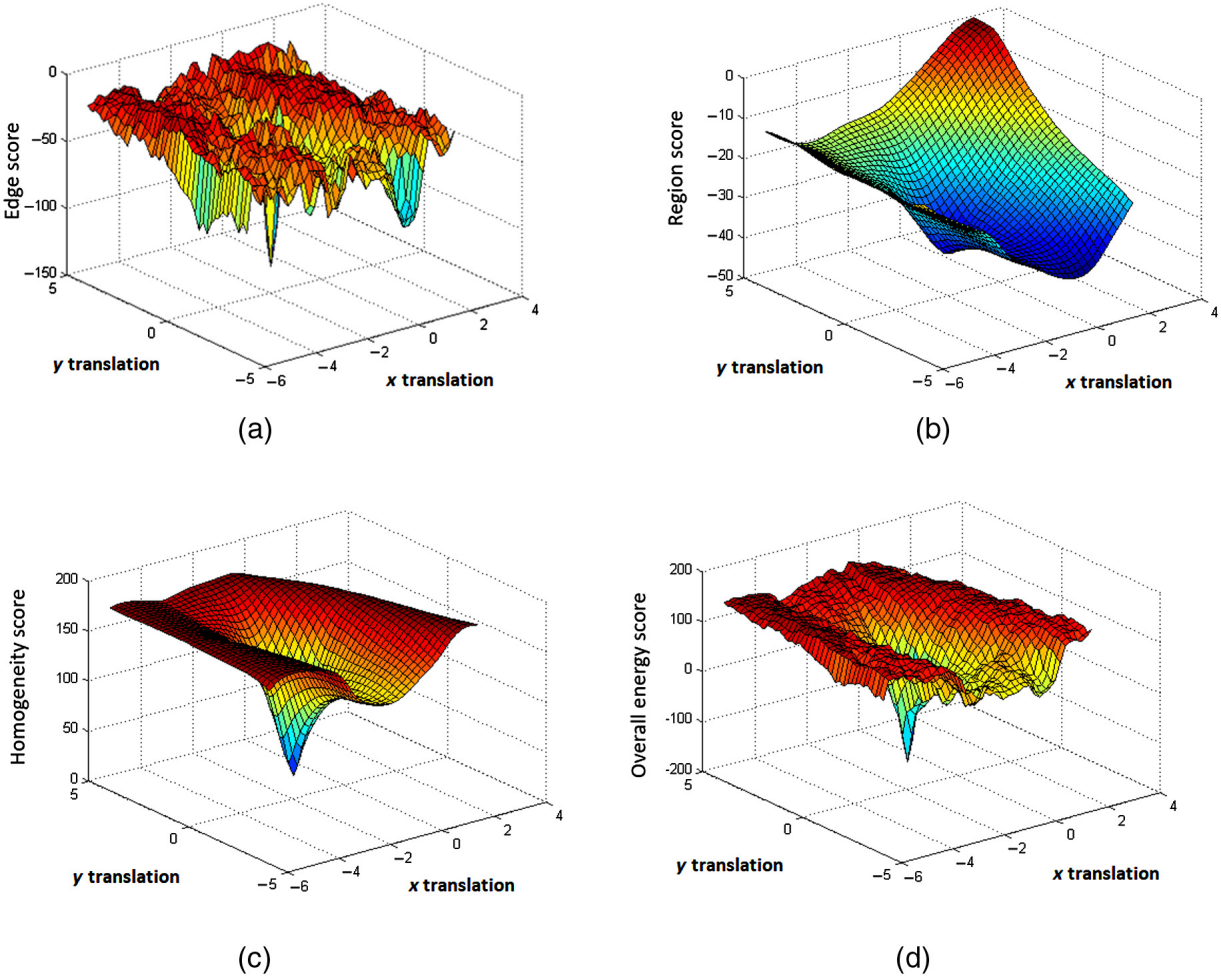
Energy function versus x axis and y axis translation from the true pose: (a) edge score, $E_{e}$; (b) region score, $E_{r}$; (c) homogeneity score, $E_{h}$; and (d) overall hybrid energy function.

It is time-consuming to use a standard collision detection library. An effective solution is achieved using part of the meshes so that only adjacent surface patches of neighboring meshes are involved by the prior knowledge that collision between femur and tibia occurs only in the femur condyle and tibial plateau. Therefore, computational complexity is significantly reduced by searching only in the surface meshes that potentially show an intersection with the surface of a neighboring mesh. We detect a collision by comparing the distance of each tibial plateau vertex to tibial bottom plane h1 with the distance of the corresponding femur condyle vertex to tibial bottom plane h2 as shown in Fig. 5. Therefore, this algorithm is independent on the flexion of the femur or tibia. The proposed collision detection algorithm is summarized in Fig. 6. Note that $E_{c}$ does not generally prohibit collisions, but makes points that lie inside other meshes less attractive. A plot of each term and the overall energy function with respect to deviation from the correct pose is shown in Fig. 7.

Figure 7(a) illustrates that there are many local minima for the edge score. This is due to the multiple matched poses of the 3-D model and the edge image. The region score in Fig. 7(b) is smoother; however, it has a relatively shallow global minimum. The homogeneity score in Fig. 7(c) has a sharp global maximum, which corresponds to the match between the 3-D model projection and the 2-D x-ray image. Figure 7(d) depicts the overall hybrid energy function. The inclusion of an edge score, region score, homogeneity score, and collision detection score results in a hybrid energy function that is relatively smooth and has an obvious global minimum.

## 3 Experiments

The training data came from the same database as our previous studies.^21^ Computed tomography (CT) scans of the knees were performed in the range from 120-mm proximal to the joint to 120-mm distal to the joint. The bones were scanned at 1- to 2-mm intervals and the volumetric data were interpolated at each 0.5 mm in the transverse plane. Automatic segmentation of the CT image was performed according to our previous study.^31^ The bone atlas was created using a method previously developed by our research group.^20^^–^^23^ Segmented three-dimensional surface mesh models were added to the atlas by adaptation of a template mesh to accurately match an input training model. This adaptation generates accurate correspondence of surface vertices.

**Fig. 8:**
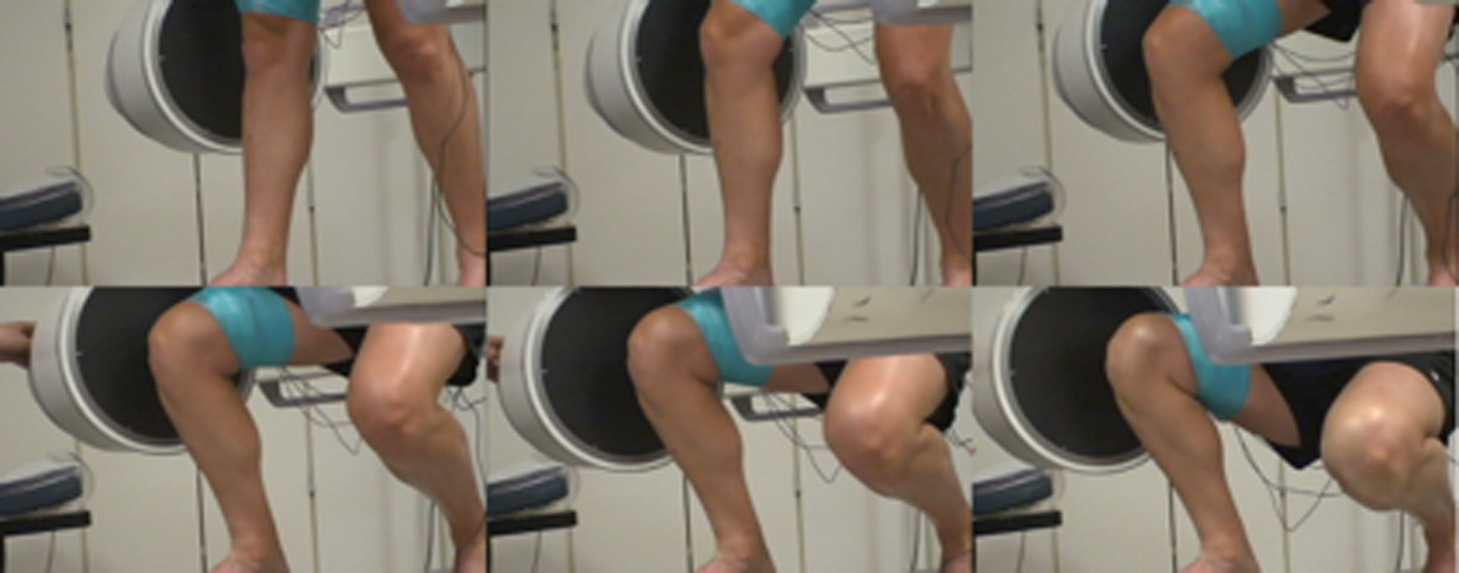
Monoplane fluoroscopic x-ray system for DKB.

The fluoroscopic x-ray system is shown in Fig. 8. Single-plane fluoroscopic x-ray sequences of DKB were acquired for five healthy subjects using a high-frequency pulsated video fluoroscopy unit with an image resolution of 640×480  pixels at 60 Hz. Because the largest knee motion occurs in the flexion and antero-posterior translation, the fluoroscopic x-ray imaging was performed in the sagittal plane as shown in Fig. 8. A perspective projection model is used for the fluoroscope by modeling the fluoroscope machine as an x-ray point source and a planar image receptor where the image is formed. The calibration procedure to remove the “pincushion” or “barrel” effect visible in the raw fluoroscopic image is described in our previous work.^13^

All subjects underwent a CT scan of the knee and triangulated CT segmentations (extracted with Avizo 8.1.0, FEI Visualization Sciences Group, Hillsboro, Oregon), segmentation was performed by thresholding the volume using a bone threshold. Slices were then examined to correct the segmentation by matching the bone contour to the boundary of the cortical bone. Output models were used as ground truth shapes. The ground truth pose was derived by 2-D–3-D registration of the ground truth shape to the fluoroscopic x-ray images with manual fitting as the initial pose using the software developed in our previous work.^13^ The training data in the atlas did not include any of the testing bones related to the five subjects of the fluoroscopic x-ray images.

Two evaluation measures were used for reconstruction and rigid registration, respectively. The reconstruction accuracy was evaluated by the root mean square (rms) error between the reconstructed shape and the ground truth one. Let A be the set of estimated shape and estimated pose points and B be the set of ground truth shape and reference pose points. Supposing they are represented as point sets $A=\{a_{0},a_{1},\ldots ,\;a_{n-1}\}$ and $B=\{b_{0},b_{1},\ldots ,b_{n-1}\}$, we define rms distance as follows:

$$\mathrm{RMS}(A,B)=\sqrt{\frac{\sum_{i=0}^{n-1}{\left(a_{i}-b_{i}\right)}^{2}}{n}}.$$ (19)

To evaluate the best achievable accuracy of the proposed KPCA-based SSM, we calculated the rms error for 3-D–3-D reconstruction of the SSM to the ground truth shape for the femur. The best achievable accuracy for the femur would be ∼0.6 to 0.7 mm.

The registration accuracy was evaluated by the motion difference with respect to the reference standard in the camera reference frame as defined in Fig. 4. Absolute mean and standard deviation of the motion differences (rotation and translation) between the tracked model and the reference standard were reported. The translation parameters relate the translation of the registered model relative to its starting position in mm, and the rotation parameters relate the angles along the defined coordinate system in degrees from the starting pose.

## 4 Results

### 4.1 Experiment 1

To demonstrate the ability of the proposed KPCA-based SSM reconstruction of femur and tibia in single-plane fluoroscopic x-ray frames, non-rigid 2-D–3-D registration was conducted on a sequence of closely spaced 2-D fluoroscopic x-ray images (around 600 fluoroscopic x-ray images) for five subjects during DKB. Both shape and pose parameters were initialized before reconstruction to prevent falling into local minima in optimization.^26^^,^^32^^,^^33^ The initial shape of the 3-D model was the mean model in the nonlinear SSM. The initial pose of the 3-D model was determined by a template-matching method.^26^

The reconstruction accuracy, listed in Table 1, was calculated by comparing the reconstructed model to the ground truth model segmented from CT.

**Table 1:** Reconstruction accuracy using single-place fluoroscopy of femur and tibia for five subjects

Subject	Femur rms error (mm)	Tibia rms error (mm)
1	1.14	1.33
2	1.81	1.25
3	1.12	1.01
4	0.92	0.92
5	0.96	1.23
Average	1.19±0.36	1.15±0.17

The rigid registration accuracy of the reconstructed models to the fluoroscopic x-ray images is summarized in Table 2, where Tx, Ty, and Tz are the mean absolute errors of translations in (x,y,z) axes and Rx, Ry, and Rz are the mean absolute errors of rotations in (x,y,z) axes as defined in Fig. 4.

**Table 2:** Pose estimation accuracy of femur and tibia.

Translation/rotation	Femur mean absolute error	Limits of agreement	Femur mean absolute error	Limits of agreement
$T_{x}$ (mm)	0.78±0.62	0.45±1.90	1.10±0.71	0.07±2.37
$T_{y}$ (mm)	0.74±0.72	−0.27±1.96	1.25±1.05	−0.40±1.59
$T_{z}$ (mm)	1.41±0.47	0.07±3.84	1.30±1.61	0.68±3.72
$R_{x}$ (deg)	0.97±0.68	0.59±2.17	0.97±0.82	−0.31±2.39
$R_{y}$ (deg)	1.05±1.06	0.68±2.74	0.90±0.81	−0.05±2.25
$R_{z}$ (deg)	0.81±0.64	0.56±1.72	0.84±0.88	−0.12±2.35

**Fig. 9:**
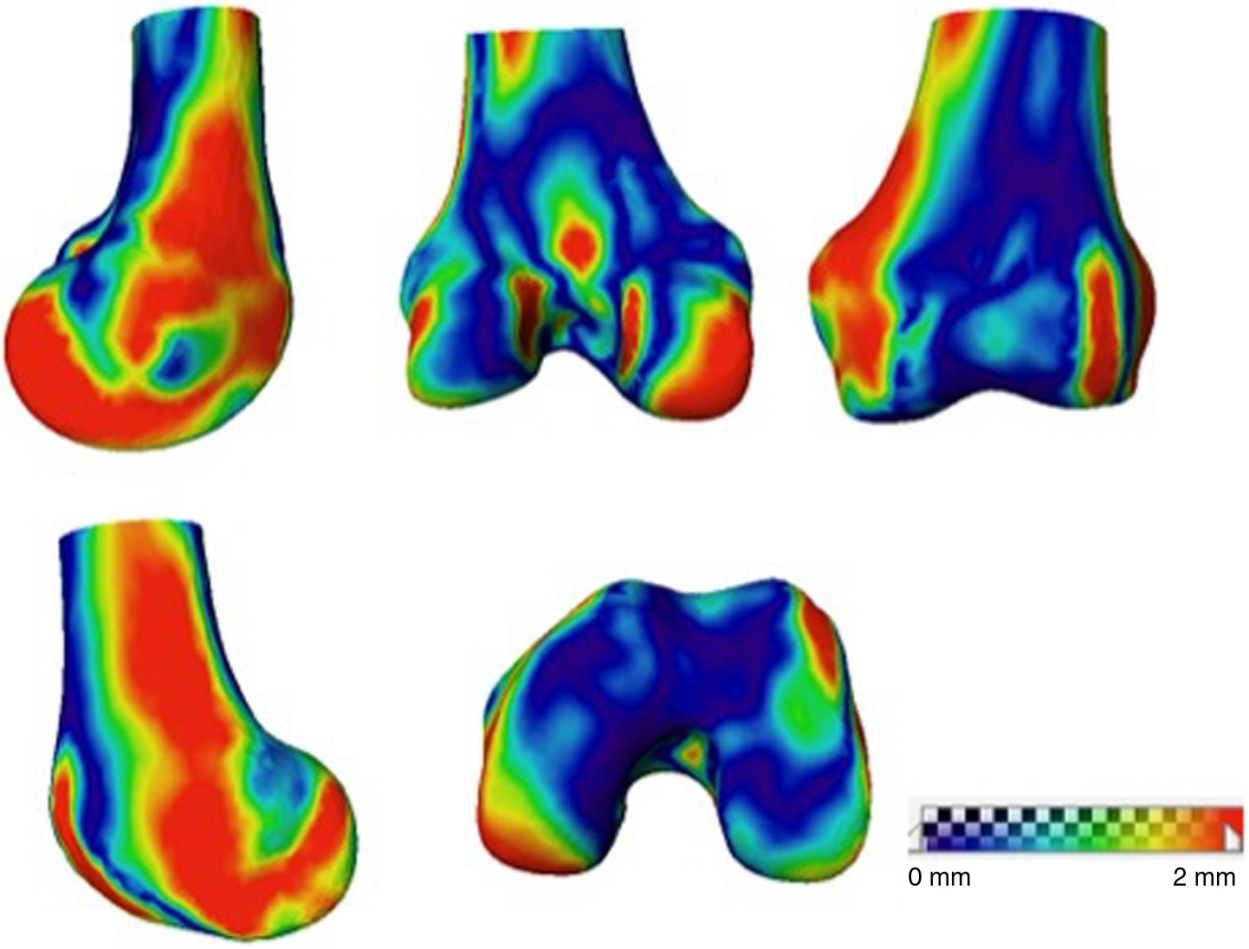
Femoral surface distance map between 3-D reconstruction and the CT model.

**Fig. 10:**
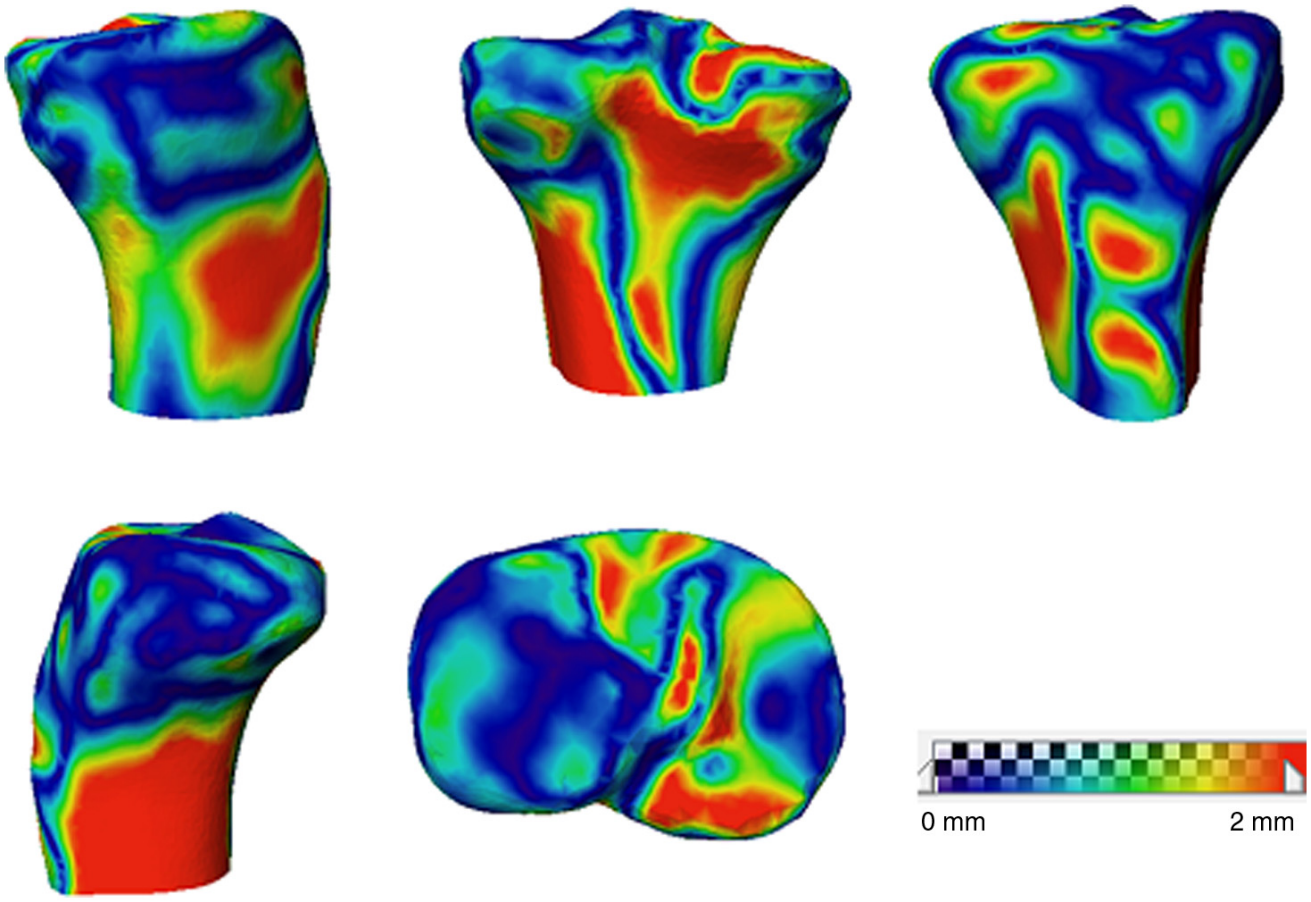
Tibial surface distance map between 3-D reconstruction and the CT model.

The surface distance maps for the reconstructed femur and tibia of subject 4 compared with the ground truth shape are shown in Figs. 9 and 10, respectively.

**Fig. 11:**
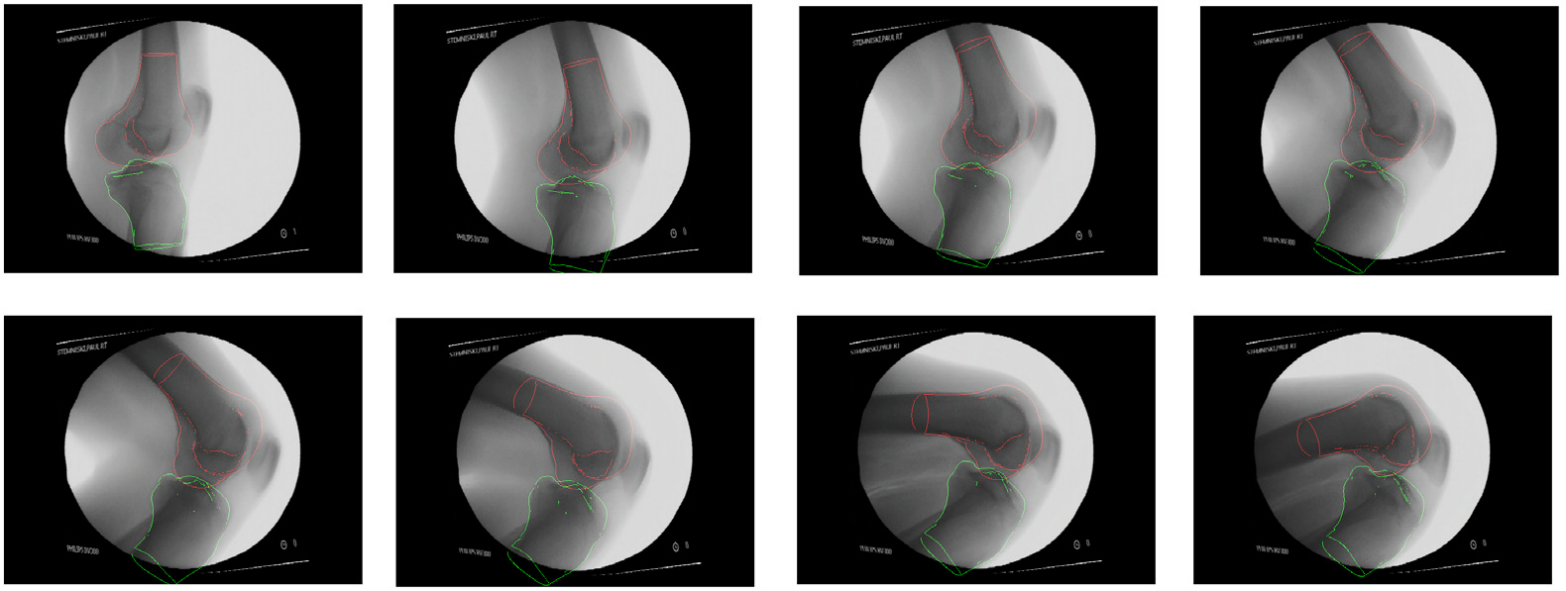
3-D model edge overlay on the x-ray images in key poses for femur and tibia during DKB (subject 4).

**Fig. 12:**
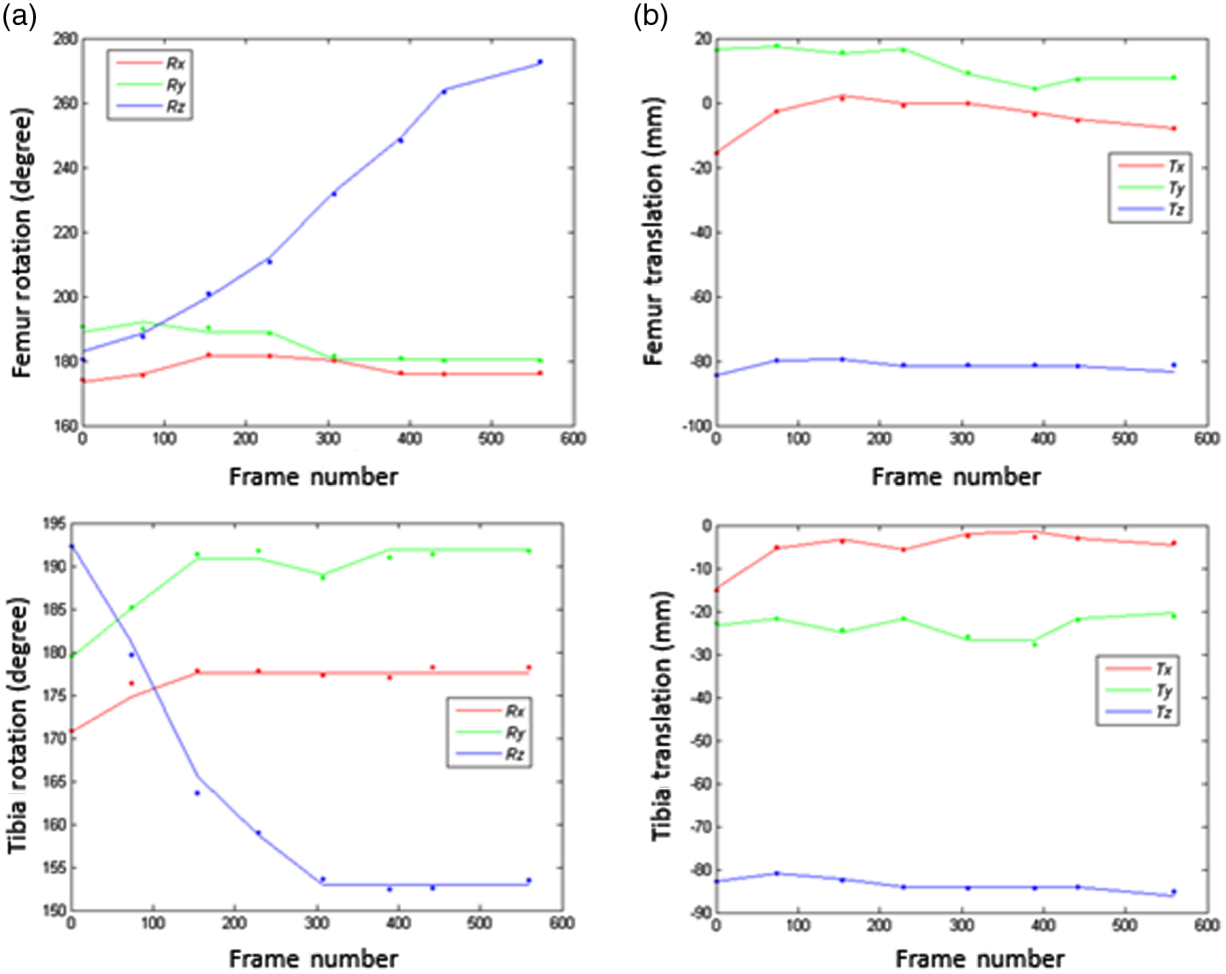
Femur and tibia kinematics of subject 4. (a) Femur and tibia rotation; (b) femur and tibia translation. Solid lines represent KPCA-based SSM fitting results and solid circles represent ground truth pose from manual fitting.

The overlays of the projected 3-D model edges on the fluoroscopic x-ray images of the same subject are shown in Fig. 11 and its motion curve is shown in Fig. 12. The solid line represents the SSM fitting results, and the dots are ground truth pose in eight selected frames. The rotation error is very small during the whole sequence of DKB with the exception of early stage of DKB. The wide spread of the z axis rotation (around the medial–lateral axis) during DKB leads to large variation in that axis for both femur and tibia. In contrast, the rotation around the x axis [anterior–posterior (AP) axis] and y axis (proximal–distal axis) is relatively steady.

### 4.2 Experiment 2

To compare single-plane reconstruction with biplane reconstruction, an additional AP view image was added to the fluoroscopic x-ray sequence. The AP view image was DRR using CT of the corresponding patient with known poses using the framework developed in our previous work.^13^ We tested all five patients, reducing the femoral average reconstruction error from 1.19 to 1.04 mm and the tibial average reconstruction error from 1.15 to 1.03 mm, as shown in Table 3.

**Table 3:** Reconstruction accuracy using biplane reconstruction of femur and tibia for five subjects

Subject	Femur rms error (mm)	Tibia rms error (mm)
1	1.09	1.22
2	1.58	1.24
3	0.96	0.89
4	0.72	0.78
5	0.87	1.04
Average	1.04±0.33	1.03±0.19

**Fig. 13:**
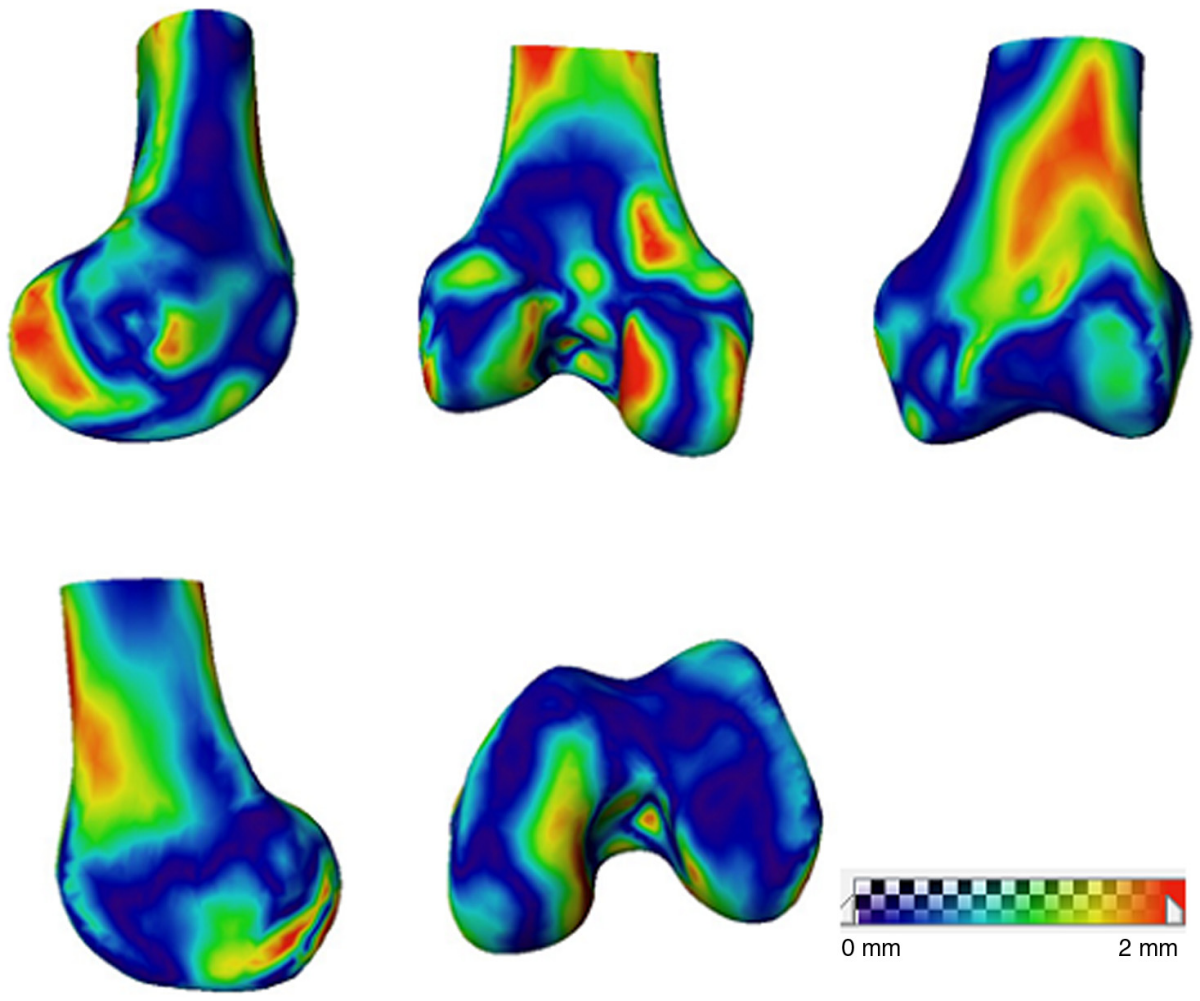
Femoral surface distance map between 3-D reconstruction and the CT model with an additional simulated AP view image (subject 4).

**Fig. 14:**
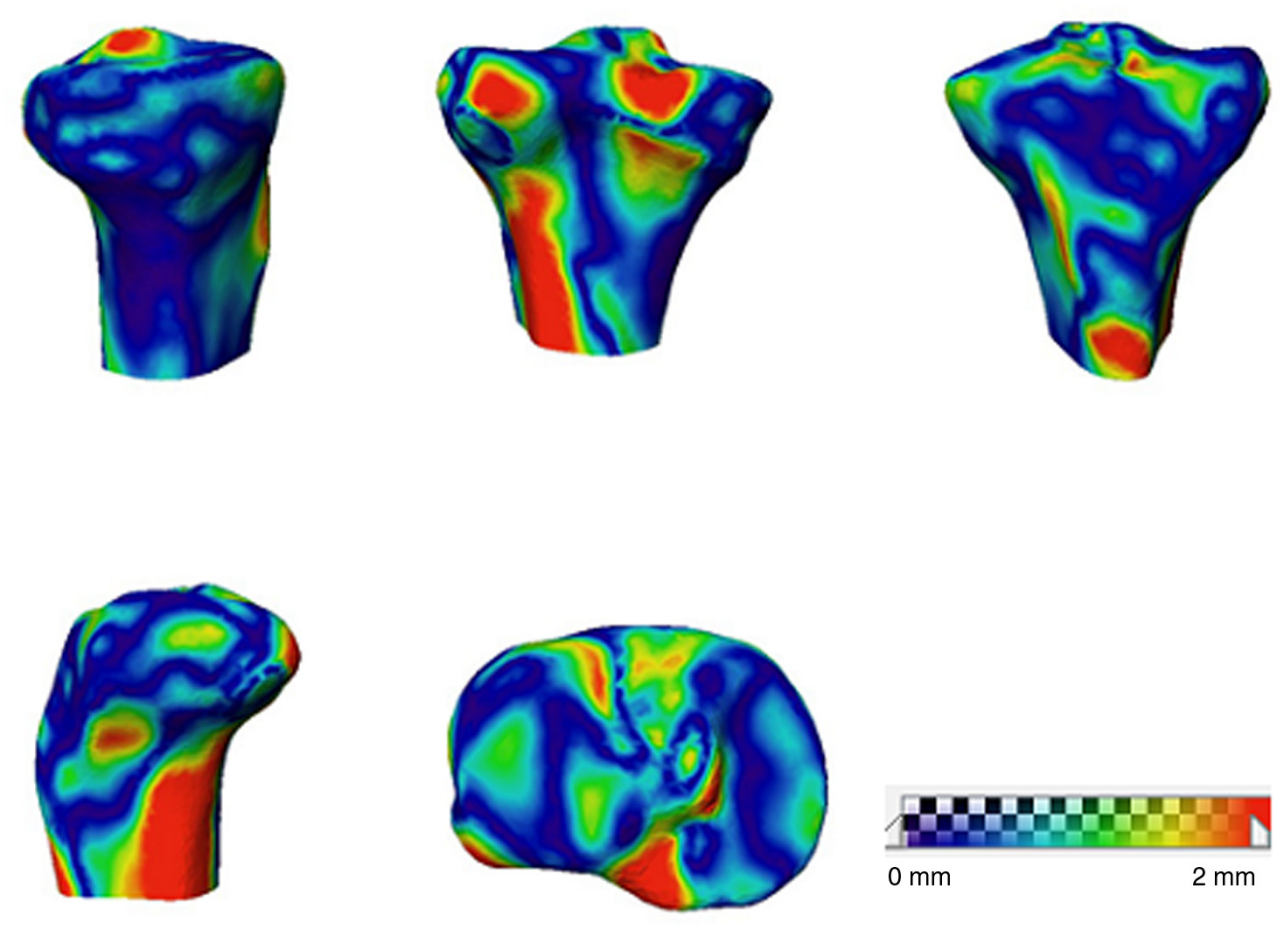
Tibial surface distance map between 3-D reconstruction and the CT model with an additional simulated AP view image (subject 4).

The surface distance maps for the reconstructed femur and tibia of subject 4 compared with the ground truth shape are shown in Figs. 13 and 14, respectively.

### 4.3 Experiment 3

**Fig. 15:**
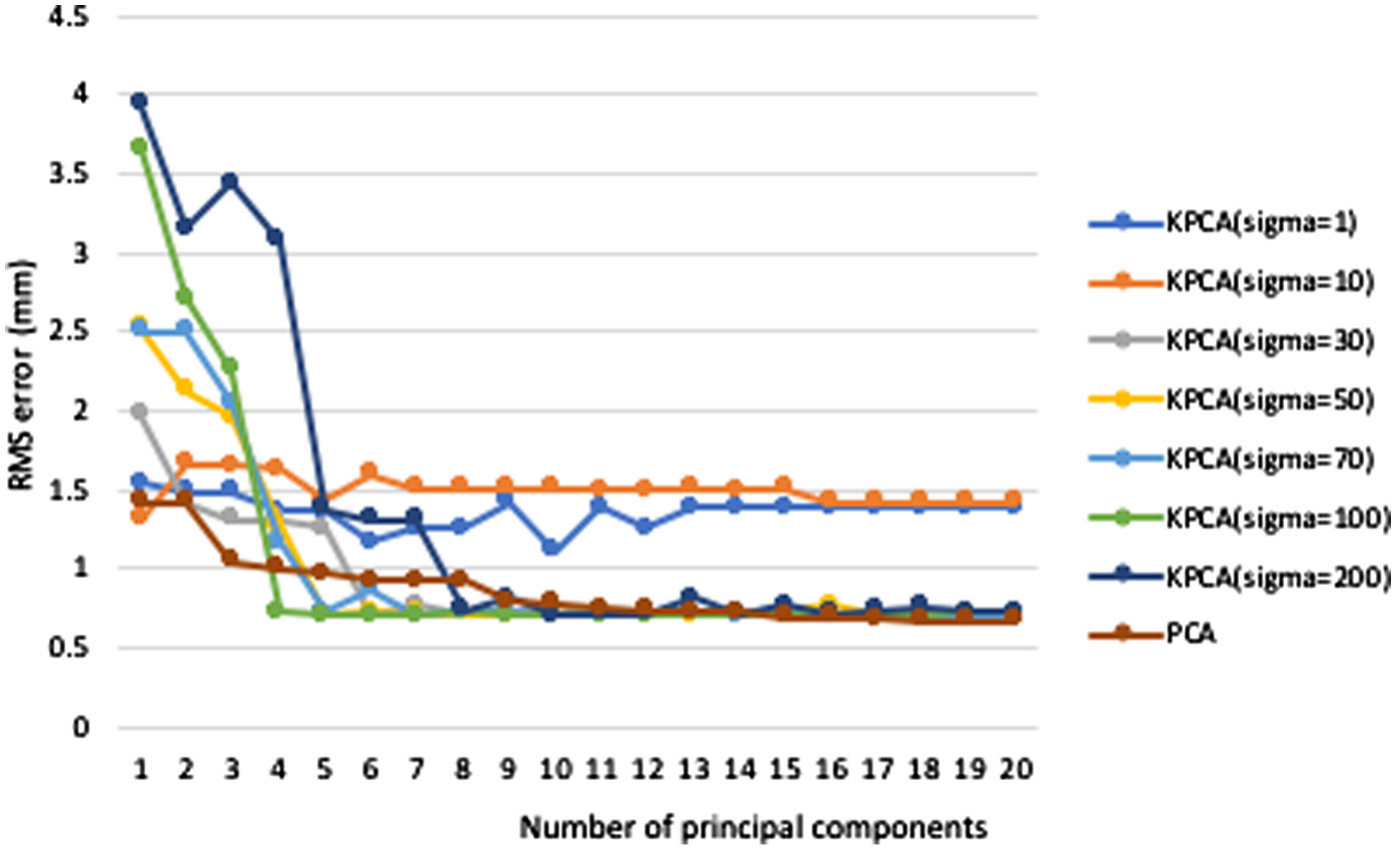
Reconstruction error of KPCA and PCA models. The KPCA curve is shown for seven different values of kernel parameter σ.

We performed leave-one-out experiments on the proposed method to compare reconstruction results between KPCA and linear PCA with different numbers of principal components (PC) σ. The reconstruction accuracy is calculated by rms error for the reconstructed surface shape compared with the ground truth. As shown in Fig. 15, the rms error decreases more quickly with the increase of σ at small numbers of principal components (pc=1 to 10), except bounding cases with large or small σ values. The rms error of σ=200 fluctuates greatly due to the decreased numerical stability for large σ value. As the number of principal components exceeds 10, the rms error stays relatively constant with increasing number of principal components. For a constant number of principal components between 5 and 10, the reconstruction error of KPCA (σ=30, 50, and 70) is smaller than that of PCA and the rms error of PCA approaches the rms error of KPCA with more than 10 principal components.

**Fig. 16:**
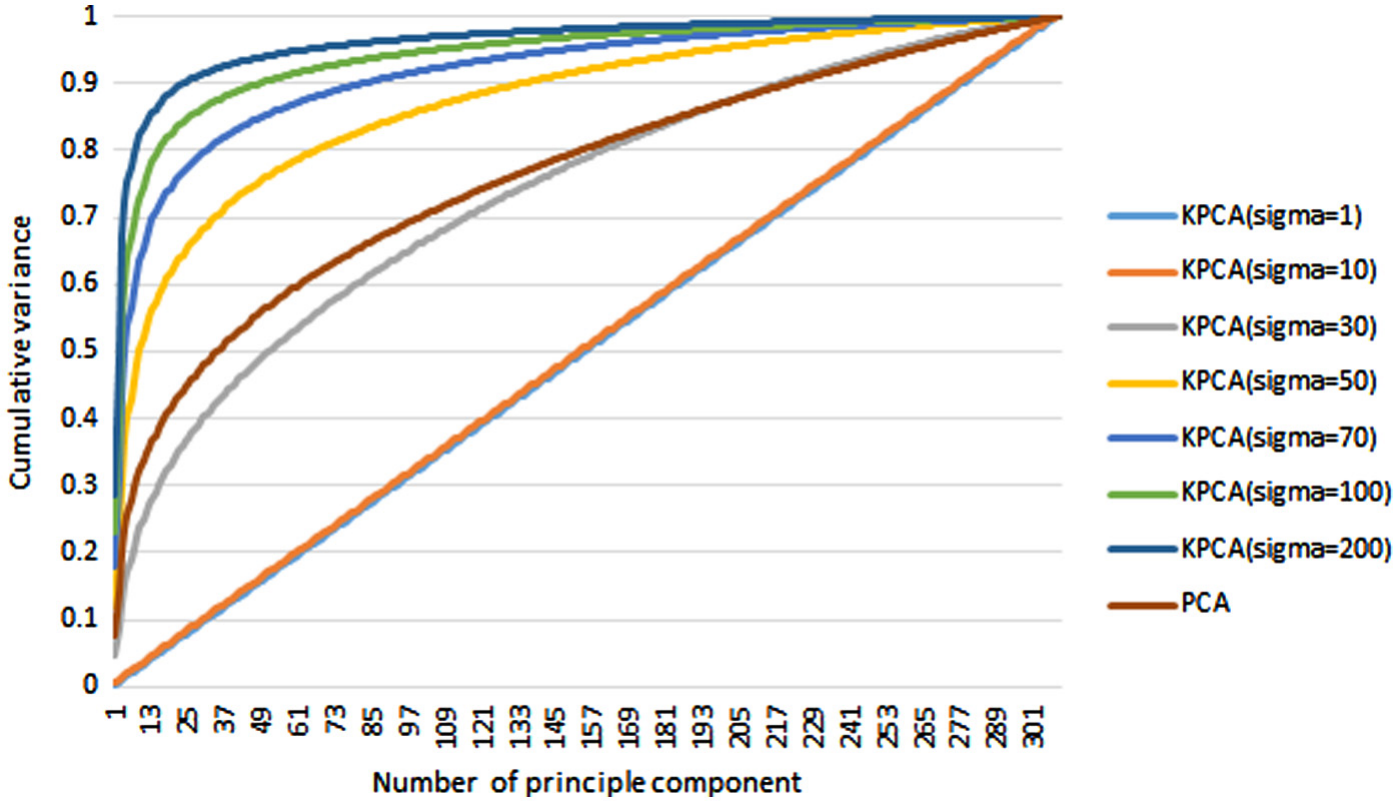
Compactness of the statistical models. Percentage of total shape variance versus the number of principal components for SSMs using PCA and KPCA.

The compactness of the model is examined by mapping the cumulative shape variance versus the number of principal components used in PCA and KPCA, as shown in Fig. 16. The cumulative variance of KPCA with different kernel parameters (σ=1, 10, 30, 50, 70, 100, and 200) is compared with that of PCA. As expected, the compactness of KPCA increases as the kernel parameter σ increases, and it approaches a straight line as σ approaches 1. Given a fixed number of principal components, a higher value of σ corresponds to a higher cumulative variance. This is because the Gaussian distribution is wider for a higher value of σ, resulting in more information remaining in the kernel mapping. The trade-off, however, is that a higher value of σ decreases the difference between different data points, decreasing the numerical stability of the KPCA reconstruction.

## 5 Discussion

We introduced an automatic method for reconstructing both femur and tibia in single-plane fluoroscopic x-ray sequences without the acquisition of prior 3-D imaging. A nonlinear SSM, KPCA, was used for 3-D reconstruction. To validate the proposed method, we report reconstruction results for five subjects conducting DKB sequences. To compare with biplane reconstruction, we added a simulated AP view image for the reconstruction, which significantly reduced the reconstruction error.

The proposed nonlinear SSM-based tracking and reconstruction method has an accuracy of 1.19±0.36  mm for femur and 1.15±0.17  mm for tibia as reported in Table 1. The surface distance map in Fig. 9 illustrates that large errors in femoral 3-D reconstruction occur in the areas that are not visible in 2-D images, such as intercondyle fossa, medial condyle, and the popliteal surface. Error in these areas is not clinically important to overall sizing and PSIs.^9^ Similarly, large errors in tibial 3-D reconstruction occur in the medial epicondyle and tibial plateau (illustrated in Fig. 10). To prove this assertion, experiment 2 was conducted by adding an AP view image to the single-plane sequence with DRR image to mimic biplanar fluoroscopic x-ray, reducing the average reconstruction error for the femur from 1.19 to 1.04 mm and for the tibia from 1.15 to 1.03 mm.

In rigid registration, the largest errors in femur poses occur in the y axis rotation (around the proximal–distal axis), as reported in Table 2. Because the bone surface has a relatively short distance to this axis, rotations around this axis cause only small changes in the contour shapes. In addition, the shape variation from the SSM may also compensate for this change in y axis rotation. The largest translation error occurs in the out-of-plane translation, because translations in this axis only cause small changes in the contour scale instead of shape changes.

Our reconstruction accuracy is comparable to single-plane or biplane reconstruction results in the literature, despite the fact that our study used the lower resolution fluoroscopic x-ray images. Of the few single-plane reconstruction studies in the literature, Baka et al.^11^ reported a biplane reconstruction rms error of 1.48 mm on 10 *in vivo* reconstructions of the distal femur from fluoroscopic x-ray sequences. Whitmarsh et al.^34^ reported an accuracy of 1.22 mm mean error of 30 femoral reconstructions from single-plane simulated dual-energy x-ray absorptiometry (DXA) images. Zheng^35^ reported an average error of 1.6 mm on a pelvic reconstruction from a single standard AP x-ray radiograph. More studies are conducted on biplane reconstruction in the literature. Laporte et al.^36^ reported a biplane reconstruction mean error of 1 mm on eight distal femurs of dry cadavers. An average mean reconstruction error of 1.2 mm was reported by Zheng et al.^37^ using two views for cadaveric femurs.

Although in this work we present a method to reconstruct the 3-D knee anatomy from single-plane fluoroscopic x-ray sequences, the method can be applied to a multi-view reconstruction as well. A multi-view reconstruction usually achieves higher reconstruction accuracy; however, multi-view fluoroscopic x-ray machines are more expensive and less used in clinics than single-view devices. Additionally, multi-view fluoroscopic x-ray machines may constrain the patient’s motion.

Reconstruction was implemented in C++ on a 3.07 GHz desktop computer with 12.0 GB of RAM. It took about 20 min to reconstruct one bone in the unoptimized implementation. Since the most time-consuming part is the shape optimization on all the frames, computation time may be greatly decreased by conducting this in parallel.

One limitation in this study is the use of a healthy population for model reconstruction. As a result, emphasis is put on the reconstruction accuracy rather than pathology analysis. Osteoporosis might result in a more varied bone shape and impact the reconstruction accuracy, thus making the reconstruction more challenging. In addition, DKB and many other activities may not be feasible in pathological patients. In the future work, we will perform reconstructions on both healthy and osteoporotic patients and on a significantly larger patient population. Collision detection on osteoporotic patients will be conducted in the future study as well. This paper is also more focused on algorithm development rather than clinical study, and we will perform clinical study and kinematics analysis with the fluoroscopic x-ray images in the future.

## 6 Conclusion

In conclusion, we present an automatic 3-D reconstruction scheme for reconstruction of knee anatomy from single-plane fluoroscopic x-ray sequences based on a nonlinear SSM. Both distal femur and proximal tibia were reconstructed from the single-plane fluoroscopic x-ray sequence during kinematic activity (e.g., DKB). The proposed method demonstrates great utility in successful elimination of prior 3-D imaging and reduction of manual labor and radiation dose on patient. It also provides more accurate information of the patients in motion than static imaging methods such as static x-ray imaging. The validation results demonstrate the reliability of the proposed method for 3-D bone model reconstruction during DKB with the average reconstruction accuracy of 1.19±0.36  mm for femur and 1.15±0.17  mm for tibia. The proposed method is promising for applications in medical interventions such as patient-specific arthroplasty design, surgical planning, surgical navigation, understanding anatomical and dynamic characteristics of joints, and characterizing joints in motion.

## 7 Appendix: Justification for Nonlinear SSM

**Fig. 17:**
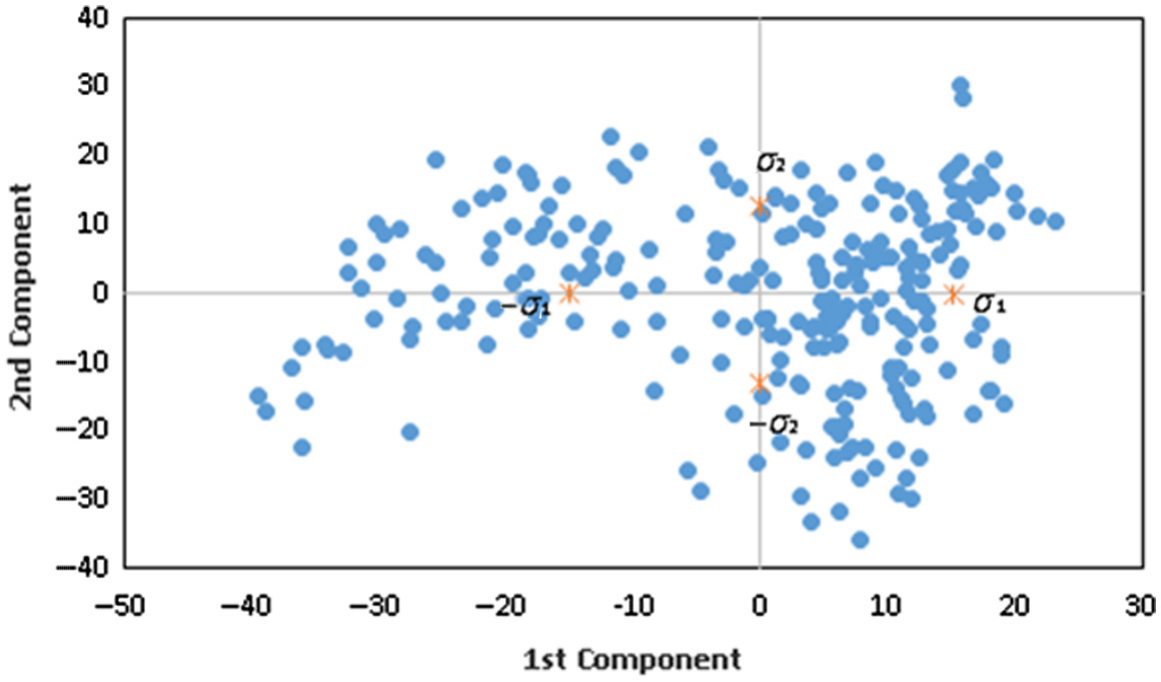
First and second principal components in the shape. Each point represents the value of the first and second principal component of PCA in the training dataset.

The linear SSMs using PCA are limited in their applicability to more complicated shape deformations, such as the knee. If the training data form distinct clusters in shape space or if the shapes are not Gaussian-distributed, the linear shape prior tends to mix classes and blur details of the shape information; consequently, the resulting shape prior no longer effectively models the shapes in the training data. We thus analyze the distribution of the training data with linear PCA. The training data are projected onto the first and second principal components of PCA. The distribution of the second principal component projection versus the first principal component projection in Fig. 17 clearly shows distinct clusters in left and right quadrants. Note that if the training set were Gaussian-distributed, then all projections should be Gaussian-distributed as well. Therefore, the training dataset is not represented by a Gaussian distribution.

**Fig. 18:**
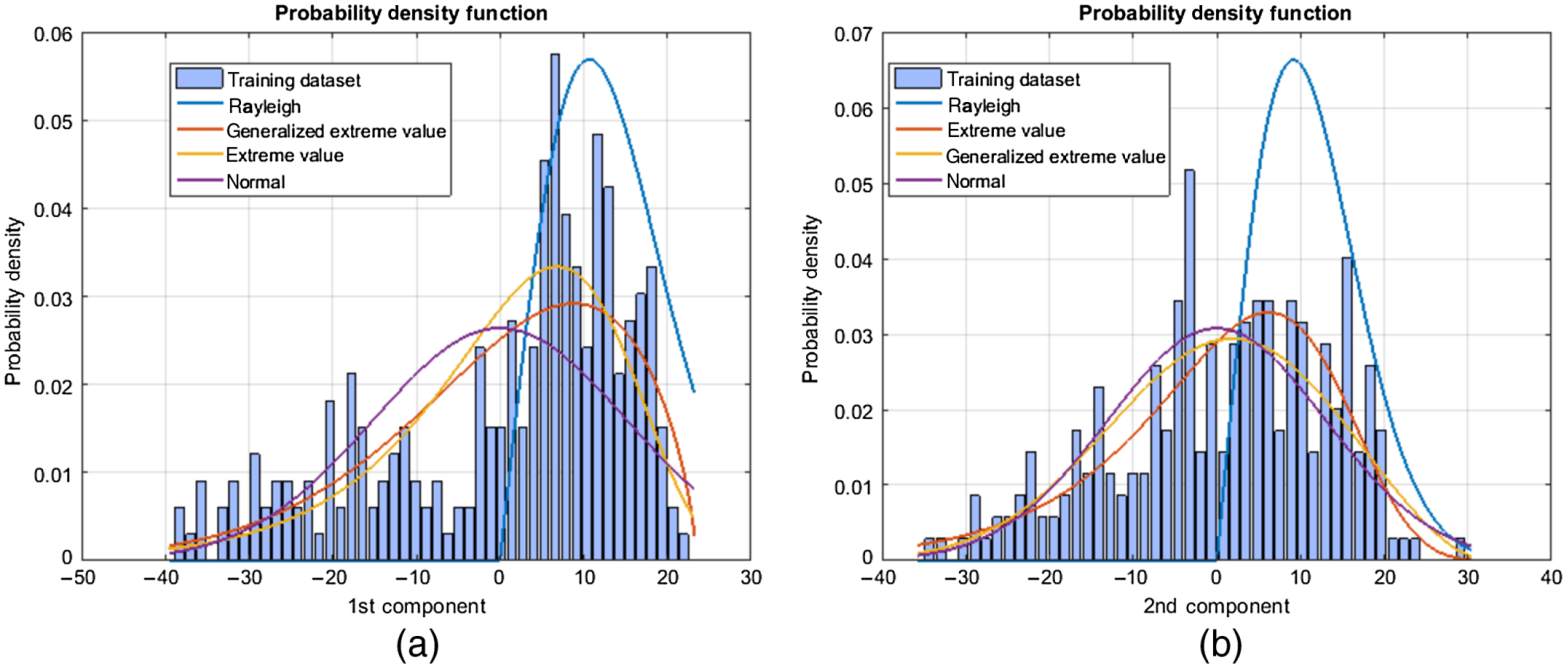
Distribution of the training dataset. Fit of various parametric probability distributions to (a) the first principal component and (b) second principal component of a training dataset for a PCA (linear) SSM.

To validate this assumption, multiple parametric probability distributions to the first and second principal component projections of the training data were performed. Results show a large gap in the probability density function (PDF) to a Gaussian distribution (Fig. 18). The assumption of Gaussian distribution has some limitation to represent the distribution of the training dataset. Therefore, kernel PCA (KPCA) is used as a nonlinear shape prior to model the knee bone shapes.
